# Effect of Sewage Irrigation on the CT-Measured Soil Pore Characteristics of a Clay Farmland in Northern China

**DOI:** 10.3390/ijerph15051043

**Published:** 2018-05-22

**Authors:** Xiaoming Guo, Tongqian Zhao, Lin Liu, Chunyan Xiao, Yuxiao He

**Affiliations:** 1 Institute of Resources & Environment, Henan Polytechnic University, Jiaozuo 454003, China; guoxiaoming@hpu.edu.cn (X.G.); xiaochunyan@hpu.edu.cn (C.X.); heyuxiao@hpu.edu.cn (Y.H.); 2 Nanjing Center, China Geological Survey, Nanjing 210016, China; liulincug@gmail.com

**Keywords:** soil pore, computed tomography, soil properties, sewage irrigation

## Abstract

Sewage irrigation has a strong influence on the physical, chemical, and biological properties of soil. However, the effects of sewage irrigation on the pore characteristics of soil are not well understood. This study compares the effects of sewage irrigation and groundwater irrigation on computed tomography (CT)-measured pore parameters and examines the relationships between CT-measured pore parameters and soil physicochemical and microbial properties. Intact soil cores were collected from S1 irrigated with sewage for 25 years, S2 irrigated with sewage for 52 years, and CK irrigated with groundwater. Various soil pore characteristics were determined, including the total pore number, macropore number (>1 mm diam.), coarse mesopore number (0.264–1 mm diam.), total porosity, macroporosity, coarse mesoporosity, and circularity. The results indicated that sewage irrigation significantly affected soil pore number and porosity. Compared with S1 and S2, CK exhibited a higher average total pore number (91), macropore number (40), coarse mesopore number (51), total porosity (2.08%), macroporosity (1.90%), and coarse mesoporosity (0.18%) throughout the 50–350 mm layer. At depths of 200–350 mm, S2 exhibited the lowest average total pore number (33), macropore number (13), coarse mesopore number (21), total porosity (0.42%), macroporosity (0.35%), and coarse mesoporosity (0.07%) among the three sites. In addition, the average pore numbers and porosity at depths of 200–350 mm decreased with increasing sewage irrigation time. There were significant positive correlations between pore features (including pore numbers and porosity) and soil properties (phosphorus content and fungi numbers). Our results suggest that decreased macropore numbers and macroporosity in the sewage-irrigated farmland may strongly intensify the accumulation of metals and nutrients in the upper layer. The findings of this study are useful for understanding the negative effects of sewage irrigation on soil pore structure and are critical for developing sustainable strategies in agriculture.

## 1. Introduction

With increasingly high demands for fresh water, sewage is being considered as a valuable resource [1]. In recent years, the use of wastewater for agricultural irrigation has become increasingly common, particularly in water-scarce areas [2,3,4,5]. Due to increasing interest in the use of sewage for irrigation and in light of the possible effects of sewage on agricultural soils and crop production, the influence of effluent irrigation on the physical, chemical, and biological properties of soil have been well documented [6,7,8]. Previous studies have shown that sewage irrigation is frequently accompanied by increases in macro- and micro-nutrients and heavy metals in the soil [9,10,11,12] in addition to changes in soil microbial functional diversity and enzymatic activity [13,14,15]. Sewage irrigation can also increase the risk of crop and groundwater pollution [16,17] and reduce soil quality [18] and the infiltration rate [8,19]. However, the effects of sewage irrigation on soil structure, particularly on pore characteristics, remain unclear.

One important feature of soil structure is the pore features, including number and size [20]. Soil pores, particularly macropores (diam. > 1000 µm), play a critical role as preferential pathways for water, air and solutes through the soil profile [21,22,23], and they have caused chemicals and contaminants to be rapidly transported into the groundwater [24]. Soil type, land use and anthropogenic activities can significantly influence the macropore characteristics [24,25]. Hu et al. [24] demonstrated that shrub encroachment resulting from anthropogenic disturbance affected soil macropores in the Inner Mongolia grassland of northern China. Zhang et al. [25] reported that the conversion of native desert soils to irrigated croplands positively influenced soil macropores in the desert-oasis ecotone. Thus, the sewage irrigation of agricultural land is expected to have a considerable effect on soil pore characteristics.

X-ray computed tomography (CT) is an imaging technique based on the computation of many transmission measurements of photographs [26]. CT has been used to determine the shape, size, orientation, and size distribution of pores at high resolutions [27]. Kumar et al. [28] quantified soil pore features, including the number of pores, number of macropores, number of coarse mesopores, porosity, macroporosity, coarse mesoporosity and fractal dimension, in agroforestry and grass buffer areas using X-ray CT. Meng et al. [23] quantified soil macropore networks under different forest communities using industrial CT in a mountainous area. Therefore, X-ray CT can be considered as a widely available method of quantifying soil pore features.

Several studies have considered the effect of irrigation with sewage on soil water permeability [8,19,29]. These studies have shown that the change in soil pores at the soil surface resulting from the swelling and dispersion of clays and accumulation of suspended solids results in a reduction in the infiltration rate or hydraulic conductivity. Although these discoveries have furthered our knowledge of soil pore features in sewage-irrigated areas, soil pore characteristics have not been interpreted clearly because of the limitations of traditional research methods. The CT technique has not yet been used to study the effect of sewage irrigation on soil structure. Furthermore, there has not been adequate research on the relationship between soil pore characteristics and soil chemical and biological properties. Therefore, the present study aimed to (1) assess the differences in the number of CT-measured soil pore features under different lengths of time of sewage irrigation (0, 25 and 52 years) and (2) correlate the CT-measured pore features with soil physicochemical and biological properties. The results of this research will provide a better understanding of the effects of sewage irrigation on soil pores and the interaction between soil pore characteristics and soil properties in the sewage-irrigated fields.

## 2. Materials and Methods

### 2.1. Study Site Description

The study site is located in the town of Luancheng (37°47′–38°01′ N, 114°29′–114°47′ E), Shijiazhuang, Hebei province, China. Shijiazhuang is one of the largest municipalities in Hebei province, with a population of over twelve million in 2013. The topography of the study site is a plain and belongs to the Hutuo alluvial-proluvial fan plain on the eastern margin of the Taihang Mountains. The study site has a semi-arid continental monsoon climate characterized by a dry and windy spring, a hot and rainy summer, a mild and cool autumn, and a cold winter [30,31,32]. The average annual temperature ranges 12–13 °C, and the annual precipitation ranges 500–600 mm. The average annual evaporation ranges 1100–1800 mm, which is approximately three times greater than the annual rainfall [30]. The soil has developed from alluvial sediment materials, and its type was mostly Mottlic Hapli-Ustic Argosols [33]. The study area is a very important region for agriculture. Conventional tillage has been commonly adopted, and most of the arable lands are under continuous winter-wheat and summer-maize rotation system [34].

This site is irrigated with sewage effluents originating from the Dongming furrow and the Xiaohe River. Sewage irrigation was initiated as early as 1950s, and the primary purpose at that time was to reuse the nutrients dissolved in sewage effluents and thus to boost the soil fertility [16,35]. Another driving force for the reuse of sewage effluents in recent times has been water resource shortages.

The sewage effluent is discharged from Shijiazhuang at an average rate of approximately 9.0 × 10^5^ m^3^ d^−1^ through the Dongmingqu canal [16], and is mainly composed of industrial wastewater and domestic wastewater. The sources of the sewage effluent mainly include pharmaceutical factories, textile mills, machinery, electronics, chemical plants, food processing plants, and domestic wastewater from household [36]. The average amounts of wastewater used for irrigation depended fundamentally on rainfall during the crop growing seasons, and they were estimated as 268 mm year^−1^ and 160 mm year^−1^, respectively [16].

Before the 1980s, the wastewater was mostly untreated water because there were few sewage treatment systems. After that, the presence of comprehensive wastewater collection and treatment systems has increased gradually with urban development. However, the treatment capacity of sewage was insufficient because of huge amounts of sewage, and the treatment systems of sewage remained low. As a result, the wastewater still had many contaminants, and the primary of pollution indicators were chemical oxygen demand, ammonia nitrogen, chlorides, lead, and chromium [36,37]. The wastewater suffered from serious pollution until 2013, after which the wastewater quality was obviously improved because the government of Shijiazhuang began to carry out the project of clean water.

### 2.2. Soil Sampling and Analysis

Two sewage-irrigated croplands and one groundwater-irrigated cropland (as the control) were selected for study. The first site (S1) has a 25-year-long history of sewage irrigation, and the second site (S2) has a 52-year-long history of sewage irrigation. The third site (CK) was considered as the control because it had never been irrigated with sewage (see Table 1). The seasonal crop for all three sites is wheat or corn.

To determine the soil pore characteristics, three replicate intact soil columns were collected at depths of 0 to 400 mm using PVC cylinders (inner diameter of 105 mm, height of 400 mm and thickness of 3 mm) at the three sites in August 2010. One additional soil column was collected to produce an artificial pore, which was used to analyze the threshold in the image analysis. Before soil sampling, the litter was completely removed to reveal bare soil. The PVC cylinders were inserted by pounding with a rubber hammer, carefully and vertically into the soil, after which they were slowly dug out using a shovel. After soil sampling, two PVC caps and packaging tape were used to retain the soil inside the cylinders, and then, the columns were sealed in plastic bags and carefully transported to the laboratory to avoid mechanical disturbance and evaporation.

To investigate the characteristics of the soil properties, disturbed samples and undisturbed samples were collected from the three sites. All disturbed soil samples were taken from depths of 0–20 cm and 20–40 cm using a Luoyang shovel, with three replicates at each site. Each replicate consisted of six random samples and was sealed on location in a plastic bag immediately before being transported to the laboratory. These samples were air-dried for 7 days and sieved with a 2-mm screen. After that, each sample was mixed uniformly, divided into two identical parts, and stored in sealed plastic bags to await further physical and chemical testing. All undisturbed soil samples were taken from the same depth using a metal-core sampler with a volume of 100 cm^3^. After weighing to determine the bulk density, each sample was stored in an aluminum box immediately before being transported to the laboratory. These samples were kept at 4 °C in a refrigerator to await microbial quantities analysis.

The water content of the soil was determined using an oven drying method at 105 °C to achieve a constant weight, the pH was determined using pH electrodes in a soil/water (1:5) suspension, the bulk density was determined using the metal-core sampler method and the electrical conductivity (EC) was determined using a conductivity meter in a soil/water (1:5) suspension while the textural classification was determined using the bouyoucos hydrometer method [38].

The contents of organic matter (OM), total nitrogen (TN), and total phosphorus (TP) as well as the cation exchange capacity (CEC) were determined with dichromate oxidation, Kjeldahl digestion, sodium hydroxide fusional and sodium acetate extraction, respectively [39]. Both total chromium content and total lead (Pb) content were measured with acid digestion—flame atomic absorption spectrometry method [38]. The quantities of bacteria, fungi and actinomycetes were determined using serial dilution and colony counting [40].

### 2.3. Quantification of Soil Pores Using CT

Soil columns were scanned using CT (Somatom Sensation 40, Siemens Healthcare, Forchheim, Germany) at the Yangtze River Scientific Research Institute. The scan system was set to 120 kV and 300 mAs, and the scan resolution was approximately 0.264 mm. Because of resolution limits, the pores measured by this procedure may not represent total pores in a soil sample. Because the soil column at the top and bottom of the cores were likely disturbed during the field collection, transportation and placement, 50 mm of the top and bottom were abandoned, restricting the depth range of scanning to 300 mm. Each column was scanned vertically at every 0.75 mm interval and generated about 533 images of 512 × 512 pixels, 31 images of which at every 10 mm interval for the depths of 50–350 mm were selected to investigate the characteristic of soil pore. The photographs were stored for the subsequent image analysis.

**Figure 1:**
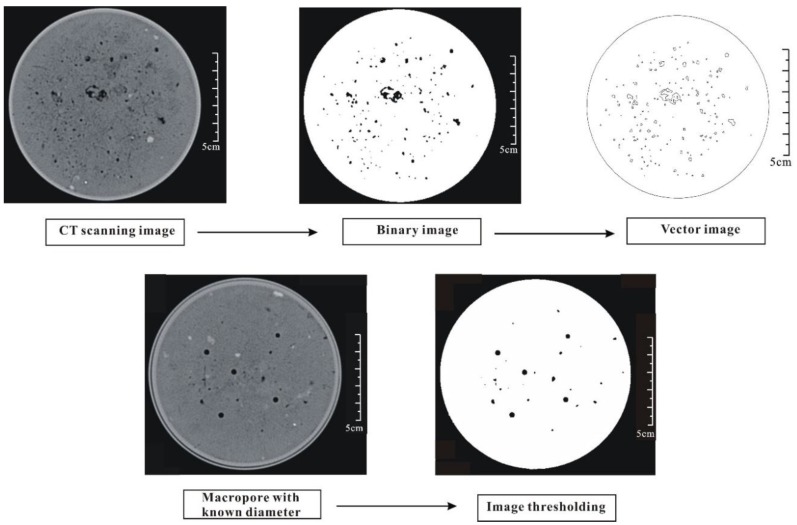
Procedures of image analysis in this study.

The images were analyzed with Photoshop CS 3 and Arc/Info 10.0 to determine the pore characteristics (see Figure 1). First, the shareholding for the sample was determined using the Photoshop software. One PVC cylinder was used to produce an artificial pore with a known diameter, and this artificial pore was scanned by CT (see Figure 1). We assumed one threshold for the sample, calculated the pore size, and compared it with the actual pore size. If the difference was excessively large, another threshold value was selected until the difference was decreased to less than 1% [24]. A value of 70 (range 0–255) was selected as the threshold value to analyze all images. After determining the threshold, the previous scanning photographs were transformed into binary images, where the black areas indicated pores and the white areas indicated soil matrix. Then, these binary images were imported into the Arc/Info software [41]. These images were changed from a shape format file to a vector file using the raster-to-polygon tool in the Arctoolbox. Finally, a DBF format file was exported according to added fields and computational geometry. The pore number, area and perimeter data were included in the file.

In this study, soil pore size was expressed by equivalent diameter for round and irregular pores. The macropore and mesopore characteristics analyzed included the total number of pores, macropore number (>1 mm diam.), coarse mesopore number (0.264–1 mm diam.), total porosity, macroporosity and coarse mesoporosity. In addition, the circularity of the pores was determined. The circularity of the pores was calculated as the product of the area of the pore and 4 π divided by the pore perimeter squared [28].

### 2.4. Statistical Analysis

Descriptive statistics (including average, minimum, maximum and coefficient of variation) for the different soil depths were used to assess the effects of sewage irrigation time on the pore characteristics. Differences in the soil pore features for S1, S2 and CK were analyzed using one-way analysis of variance (ANOVA) and a least significant difference (LSD) test. Correlation coefficients were calculated to determine the relations between the soil properties and pore features. The statistical analyses were performed using version 13.0 of the SPSS software (SPSS Inc., Chicago, IL, USA).

## 3. Results

### 3.1. Pore Number

The results of the quantitative measurement of the soil pore numbers, including total pore number, macropore number (>1 mm diam.) and coarse mesopore number (0.264–1 mm diam.), are shown in Table 2.

There is a large difference between the minimum and maximum values of the average number of total pores, macropores, and coarse mesopores, particularly for soil depths of 50–100 mm. The average total pore number and macropore number under S1 and S2 had high CV values (>0.35) for the four soil depths, indicating high spatial variability in the sewage-irrigated area.

The soil pore numbers varied with irrigated water and soil depth. The average total pore number, macropore number, and coarse mesopore number had similar vertical distributions in the three sites, decreasing with increasing soil depth. For S1, S2 and CK, the total pore number at depths of 300–350 mm decreased by 65, 89, and 65%, respectively, compared with that at depths of 50–100 mm, and the macropore number decreased by 68, 90, and 71%, respectively. The average total pore number, macropore number, and coarse mesopore number at S2 were larger than those at CK at depths of 50–100 mm. In contrast, for the depths of 100–200, 200–300, and 300–350 mm, S2 exhibited a lower average total pore number, macropore number, and coarse mesopore number than CK. Compared with S1, S2 contained a higher average total pore number, macropore number, and coarse mesopore number for the depths of 50–100 and 100–200 mm but lower numbers for the depths of 200–300 and 300–350 mm.

**Figure 2:**
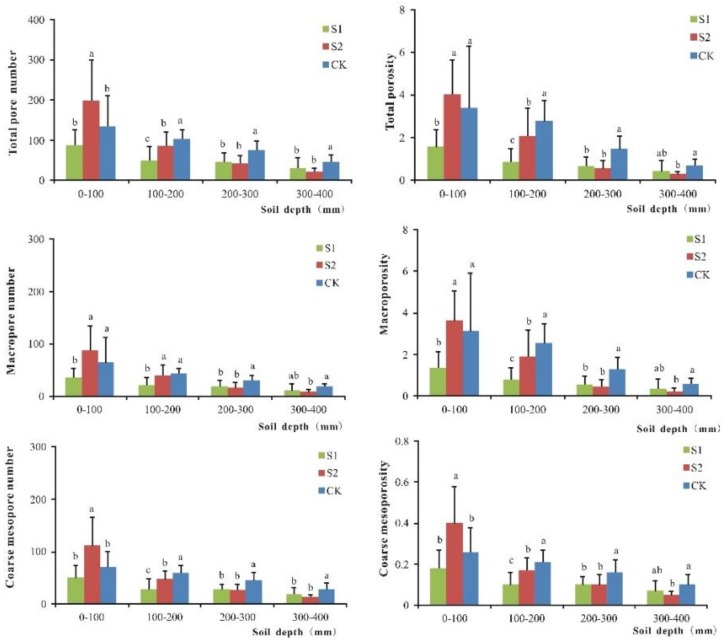
One-way analysis of variance of total pore number, total porosity, macropore number, macroporosity, coarse mesopore number, and coarse mesoporosity under S1 (irrigated with sewage for 25 years, 3 replicates), S2 (irrigated with sewage for 52 years, 3 replicates), and CK (irrigated with groundwater, 3 replicates).

For the 50–100 and 100–200 mm depths, the difference in the average numbers of total pores, macropores and mesopores between S1 and S2 was significant, whereas the difference was not large for the 200–300 and 300–350 mm depths (see Figure 2). For each of the four soil layers, the difference in the average numbers of total pores and mesopores between S2 and CK was significant, and the difference between S1 and CK was obvious for the 100–200, 200–300 and 300–350 mm depths. In addition, for the 200–300 and 300–350 mm depths, the difference in the average number of macropores between S2 and CK was significant, and for the 50–100, 100–200 and 200–300 mm depths, the difference between S1 and CK was large. In conclusion, sewage irrigation had strong effects on soil pore numbers, with the pore numbers decreasing at greater depths.

**Figure 3:**
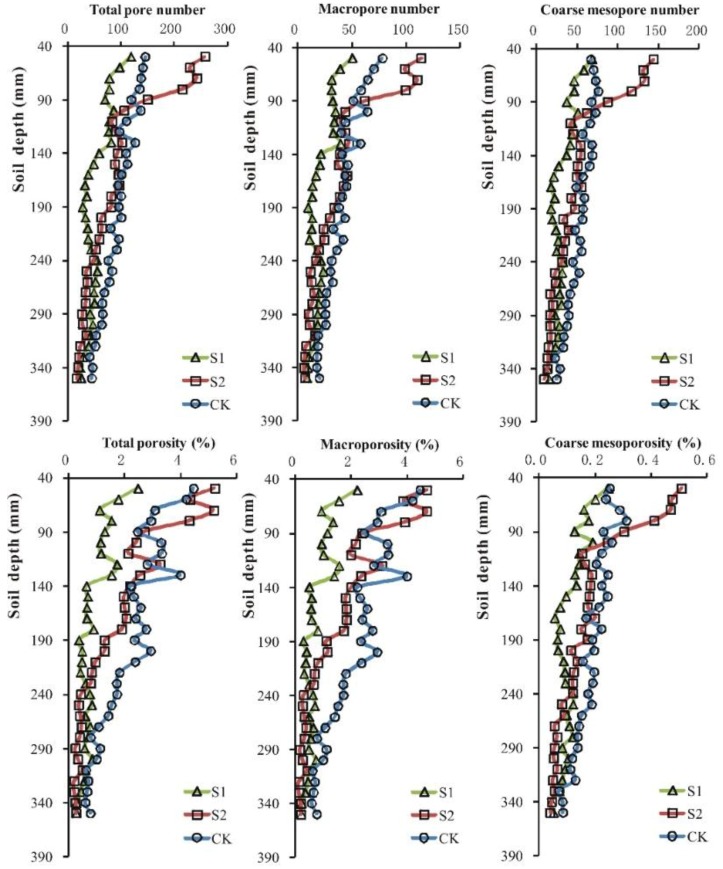
Distribution of total pore number, macropore number, coarse mesopore number, total porosity, macroporosity, and coarse mesoporosity at every 1-cm interval of the 5–35 cm depth from S1 (irrigated with sewage for 25 years, 3 replicates), S2 (irrigated with sewage for 52 years, 3 replicates), and CK (irrigated with groundwater, 3 replicates).

Figure 3 also shows similar trends for the average pore number as those in Table 2 and provides more detail regarding the numbers. The pattern for the average total pore number, macropore number, and mesopore number under S1 and CK was relatively steady, particularly for depths of 140–360 mm. In contrast, the trends fluctuated considerably under S2, particularly for depths of 50–110 mm. The average total pore number, macropore number, and mesopore number at depths of 50–90 mm were greatest at S2, followed by CK and then S1. At depths of 100–230 mm, the average total pore number, macropore number, and mesopore number varied among three sites, with the order CK > S2 > S1. For the depths of 240–350 mm, the order for the average total pore number, macropore number, and mesopore number was CK > S1 > S2. In conclusion, the average pore number decreased at greater depths as the duration of sewage irrigation increased.

### 3.2. Porosity

Table 2 shows the large difference between the minimum and maximum values of the average porosity, particularly for soil depths of 50–100 mm. The average values of the total porosity and macroporosity under S1 and S2 had high CV values (>0.35) for the four soil depths, indicating high spatial variability in the sewage-irrigated fields.

The porosity varied with irrigation water and soil depth. The average total porosity, macroporosity and mesoporosity had similar vertical distributions at the three sites, decreasing with increasing soil depth. For S1, S2 and CK, the total porosity at depths of 300–350 mm decreased by 72, 93 and 80%, respectively, compared to that at depths of 50–100 mm, whereas the macroporosity decreased by 73, 94 and 82%, respectively. The average total porosity, macroporosity and mesoporosity at S2 were larger than those at CK for depths of 50–100 mm. In contrast, for depths of 100–200, 200–300, and 300–350 mm, S2 exhibited a lower average total porosity, macroporosity, and mesoporosity than CK. Compared with S1, S2 contained a higher average total porosity, macroporosity, and mesoporosity for the 50–100 and 100–200 mm depths but a lower porosity for the 200–300 and 300–350 mm depths.

For the 50–100 and 100–200 mm depths, the difference in the average total porosity, macroporosity, and mesoporosity between S1 and S2 was significant, whereas the difference was not large for the 200–300 and 300–350 mm depths (see Figure 2). The difference in the average mesoporosity between S2 and CK was significant for each of the four soil layers, and the difference between S1 and CK was large for the 100–200 and 200–300 mm depths. In addition, the difference in the average total porosity and macroporosity between S2 and CK was large for the 100–200, 200–300 and 300–350 mm depths, and the difference between S1 and CK was large for the 50–100, 100–200 and 200–300 mm depths. The results indicated that sewage irrigation significantly affected porosity.

The vertical distributions of total porosity and macroporosity at the three sites fluctuated more than the mesoporosity (see Figure 3). The patterns for the average total porosity and macroporosity under S1 were relatively steady, particularly for depths of 140–360 mm. In contrast, the average total porosity and macroporosity fluctuated considerably under S2, particularly for the depths of 50–190 mm, as did it under CK, particularly for the depths of 50–280 mm. The average total porosity, macroporosity, and mesoporosity under the three sites at depths of 50–90 mm followed the order S2 > CK > S1. For the 100–230 mm depth, the order of the total porosity, macroporosity, and mesoporosity was CK > S2 > S1, and for the 240–350 mm depth, the order of the total porosity, macroporosity, and mesoporosity was CK > S1 > S2.

### 3.3. Circularity

Circularity is an important feature of pore shape. Table 2 indicates the rather low difference between the minimum and maximum values of circularity under S1 (0.56–0.71), S2 (0.60–0.70) and CK (0.60–0.72). In addition, the CV values of circularity under S1 (0.022–0.051), S2 (0.023–0.042) and CK (0.020–0.044) were small. In conclusion, the differences in circularity among the three sites and the variation in circularity with soil depth were rather small.

### 3.4. Relationships between Soil Pore Features and Soil Properties

A correlation analysis of pore features and soil properties (details are in the Supplementary Materials Tables S2–S4) among the three sites is provided in Table 3. Soil water content was positively correlated with various soil pore features, including total pore number (R = 0.853, *p* < 0.05), macropore number (R = 0.851, *p* < 0.05), coarse mesopore number (R = 0.853, *p* < 0.05), total porosity (R = 0.876, *p* < 0.05), macroporosity (R = 0.876, *p* < 0.05) and coarse mesoporosity (R = 0.844, *p* < 0.05), whereas it was negatively correlated with circularity (R = −0.904, *p* < 0.05). In addition, the content of total phosphorus was positively correlated with total pore number (R = 0.830, *p* < 0.05), macropore number (R = 0.836, *p* < 0.05), coarse mesopore number (R = 0.824, *p* < 0.05) and coarse mesoporosity (R = 0.836, *p* < 0.05). The fungi numbers exhibited significant positive correlations with macropore number (R = 0.816, *p* < 0.05), total porosity (R = 0.813, *p* < 0.05) and macroporosity (R = 0.812, *p* < 0.05), whereas it was negatively correlated with circularity (R = −0.923, *p* < 0.01). These results indicated that soil pores may be strongly influenced by soil properties.

## 4. Discussion

Soil pore characteristics are mainly influenced by soil type, land use and anthropogenic activity [24,25]. Luo et al. [21] observed a distinction in pore features between two soil types (silt loam and sand) and two land uses (crop and pastures). Hu et al. [24] demonstrated that shrub encroachment due to anthropogenic disturbance strongly affected soil pores in the Inner Mongolia grassland of northern China. In this study, within the same land use and similar soil type (see Table 1 and Table S2), distinctly different pore number and porosity were observed among S1, S2, and CK because of the effects of anthropogenic activity, such as sewage irrigation. Compared with S1, CK had significantly higher average pore number (including total pore number, macropore number, and coarse mesopore number) and porosity (including total porosity, macroposity, and coarse mesoporosity) for the depths of 100–200 and 200–300 mm. The similar results of pore number and porosity were seen in the comparison between S2 and CK for the depths of 200–300 and 300–350 mm. The reason of decreasing pore number and porosity in the sewage irrigated sites was mainly related to the quality of sewage effluents. Sewage effluents had higher contents of total organic carbon, potential of hydrogen, electrical conductivity, sodium ion, sodium adsorption ratio, cadmium (Cd), chromium (Cr), and lead (Pb) than the irrigated groundwater (see Table S1). High inputs of nutrients, salts and heavy metals to soils resulting from the sewage irrigation can result in biological clogging, the dispersion of soil aggregates and a reduction in root numbers [1,29,42]. These results would strongly influence the soil pore structure, particularly by reducing the number of soil pores and porosity. In addition, the circularity among three sites did not have obvious difference, as it may be mainly affected by the land use and soil type. In conclusion, sewage irrigation can significantly affect soil pore number and porosity, and it may have a slight effect on the pore shape, such as the circularity.

Soil pore characteristics are also influenced by the soil properties, particularly by organic content [21,25]. Luo et al. [21] reported a significant positive correlation between the organic content and macroporosity, and organic matter accumulated because the perennial grass produced abundant living/decaying roots. In this study, although sewage effluents contained higher organic content than the irrigated groundwater, the macropore and macroporosity in the S1 and S2 were not improved by the increasing input of organic matter into the soil. We found that there was not a significant correlation between the organic content and the macropore characteristic. The reason may be related to the source of organic matter, most of which was not from the roots but from the sewage effluents. The addition of exogenous organic matter to soil may not clearly influence the soil macropores. Our results indicated that the correlation between soil properties, including fungi numbers and total phosphorus, and macropore features was positive, suggesting that the soil macropore structure may be affected by the soil fungi numbers and total phosphorus in the fields.

Several studies have showed that the time of sewage irrigation can significantly affect the physicochemical and microbial properties of soil [43,44]. Lucho-Constantino et al. [43] reported that the relation between the contents of organic carbon, total boron, and total lead and time of sewage irrigation (6–41 years) was clearly positive. Guo et al. [44] reported that the contents of bacteria numbers, actinomycete numbers, sucrase activity and phosphatase activity were significantly correlated in a positive manner with time of sewage irrigation (0–52 years). In this study, we discussed the effects of sewage irrigation time on soil pore. We found that the difference in the average macropore number and macroporosity for the depths of 300–350 mm between S2 and CK was significant, but the difference between S1 and CK was indistinctive. There was significant difference in the average pore number and porosity for the depths of 50–100 and 100–200 mm between S1 and S2. In addition, the pore number and porosity for the depths of 240–350 mm decreased with the increasing time of sewage irrigation. Hence, the time of sewage irrigation had noticeable and detailed effects on the characteristic of soil pore number and porosity.

In this study, both macropore number and macroporosity under S1, S2 and CK decreased greatly with soil depth. For S1, S2 and CK, the macropore number at depths of 300–350 mm decreased by 68, 90 and 71%, respectively, compared to that at depths of 50–100 mm, and the macroporosity decreased by 73, 94 and 82%, respectively. The reason for the vertical distribution of macropore number and macroporosity was mainly due to different genetic mechanism of soil macropores. Biopores such as root channels are great pores and its size generally decrease with depth, whereas relatively small pores are likely inter-aggregate macropores, such as those formed by wetting and drying [21]. S1, S2 and CK at depths of 50–200 mm contained obviously greater macropores and macroporosity than that at depth of 200–350 mm, as its formative process may be associated with the crop roots or decomposed crop residues that were mainly concentrated in the plow layer (0–20 cm). In contrast, S1, S2 and CK at depths of 200–350 depths contained relatively smaller macropores and macroporosity, as its formative process may be related to alternate wetting and drying.

Although soil macropores constitute only a small percentage of total porosity, they play a critical role that is related to transport of water and solutes in the soil [22,25]. Soil macropres are the main cause for the phenomenon of preferential flow, which can reduce the adsorbing capacity of soil and residence time of solutes, and increase the transport of heavy metals and nutrients through the soil profile [24,45]. Therefore, the decreasing soil macropores in the sewage irrigated areas would reduce the transportation of heavy metals and nutrients into the deep layer of soil. In the study area, for the 0–200 mm depth, the contents of total nitrogen, total phosphorus, Cr and Pb under three sites increased with the increasing time of sewage irrigation. But for the 200–400 mm depth, the contents of total nitrogen, total phosphorus and Pb did not increase with the time (see Table S3). We speculate that the decreasing soil macropores may strongly intensify the accumulation of metals and nutrients in the upper layer.

## 5. Conclusions

Changes in soil pore characteristics were quantified using computed tomography (CT) scanning in this study. The results demonstrated that sewage irrigation significantly decreased the soil total pore number, macropore number, coarse mesopore number, total porosity, macroporosity, and coarse mesoporosity at depths of 200–350 mm. A longer duration of sewage irrigation led to a lower pore number and porosity at depths of 200–350 mm. The results of this research can improve our understanding of soil pore structure at various depths in sewage-irrigated fields. This knowledge is useful for understanding the negative effect of sewage irrigation on soil pore structure and is critical for developing sustainable strategies in agriculture.

## Figures and Tables

**Table 1:** Basic situation of soil sampling sites.

Sites	Position	Crop Type	Sewage Irrigation Time (Years)
S1	N 37°58′26.2′′, E 114°32′34.2′′	Corn	25
S2	N 37°58′, E 114°31′41.1′′	Corn	52
CK	N 37°57′36.4′′, E 114°32′30.3′′	Corn	0

**Table 2:** Descriptive statistics of soil pore feature under S1 irrigated sewage for 25 years, S2 irrigated with sewage for 52 years and CK irrigated with groundwater at the 50–100 (*n* = 18), 100–200 (*n* = 30), 200–300 (*n* = 30) and 300–350 (*n* = 15) mm depths.

Properties	Depth (mm)	S1				S2				CK			
		Mean	Max.	Min.	CV	Mean	Max.	Min.	CV	Mean	Max.	Min.	CV
Total pore number	50–100	88	165	23	0.44	200	355	70	0.50	136	292	58	0.56
	100–200	51	124	16	0.65	87	149	38	0.40	104	163	70	0.21
	200–300	47	99	14	0.47	43	84	7	0.47	77	123	45	0.29
	300–350	31	83	9	0.83	23	44	15	0.38	47	78	19	0.38
Macropore number	50–100	37	78	8	0.49	88	163	24	0.53	65	166	18	0.74
	100–200	22	59	5	0.68	40	80	14	0.53	44	66	25	0.25
	200–300	19	45	2	0.63	17	33	1	0.59	31	55	11	0.29
	300–350	12	40	1	1.08	9	22	3	0.56	19	29	10	0.32
Coarse mesopore number	50–100	51	87	15	0.44	112	192	43	0.48	71	126	34	0.43
	100–200	29	73	3	0.69	48	72	23	0.33	60	98	41	0.24
	200–300	28	54	12	0.38	27	52	5	0.45	46	77	25	0.32
	300–350	20	45	7	0.65	14	22	9	0.29	29	53	9	0.44
Total porosity (%)	50–100	1.56	3.94	0.45	0.53	4.03	6.93	1.07	0.40	3.41	10.11	0.86	0.84
	100–200	0.88	2.39	0.21	0.73	2.07	5.83	0.46	0.64	2.77	5.46	0.92	0.35
	200–300	0.66	1.60	0.10	0.67	0.55	1.42	0.05	0.71	1.46	2.59	0.43	0.42
	300–350	0.44	1.47	0.05	1.14	0.28	0.69	0.10	0.57	0.68	1.24	0.18	0.49
Macroposity (%)	50–100	1.37	3.63	0.42	0.55	3.63	6.31	0.89	0.40	3.14	9.67	0.64	0.89
	100–200	0.78	2.18	0.15	0.77	1.90	5.58	0.35	0.67	2.56	5.13	0.74	0.37
	200–300	0.56	1.45	0.04	0.73	0.46	1.26	0.03	0.76	1.30	2.45	0.30	0.45
	300–350	0.37	1.32	0.01	1.22	0.23	0.62	0.05	0.70	0.58	1.13	0.17	0.52
Coarse mesoporosity (%)	50–100	0.18	0.34	0.04	0.46	0.40	0.72	0.18	0.45	0.26	0.49	0.11	0.50
	100–200	0.10	0.22	0.01	0.29	0.17	0.28	0.07	0.35	0.21	0.35	0.12	0.60
	200–300	0.10	0.19	0.04	0.38	0.10	0.19	0.01	0.50	0.16	0.27	0.08	0.40
	300–350	0.07	0.17	0.02	0.50	0.05	0.08	0.03	0.40	0.10	0.20	0.01	0.71
Circularity	50–100	0.64	0.66	0.61	0.022	0.64	0.69	0.60	0.042	0.64	0.66	0.61	0.020
	100–200	0.65	0.71	0.56	0.051	0.66	0.69	0.63	0.023	0.64	0.68	0.62	0.022
	200–300	0.66	0.71	0.63	0.024	0.66	0.70	0.61	0.035	0.66	0.68	0.63	0.020
	300–350	0.65	0.68	0.61	0.035	0.66	0.70	0.64	0.035	0.66	0.72	0.60	0.044

Max., Min., and CV represented maximum, minimum and coefficient of variation, respectively.

**Table 3:** Correlation analysis between soil pore characteristic and soil properties among three sites.

Properties	WC	BD	CC	EC	OC	TN	TP	BN	FN	AN
TPN	0.853 *	−0.485	0.049	−0.344	0.684	0.674	0.830 *	0.737	0.808	0.681
Total porosity	0.876 *	−0.476	−0.016	−0.452	0.637	0.604	0.773	0.656	0.813 *	0.590
MN	0.851 *	−0.480	0.054	−0.336	0.693	0.679	0.836 *	0.743	0.816 *	0.687
Macroporosity	0.876 *	−0.475	−0.021	−0.460	0.632	0.598	0.767	0.648	0.812 *	0.581
CMN	0.853 *	−0.489	0.044	−0.352	0.674	0.699	0.824 *	0.730	0.800	0.674
Coarse mesoporosity	0.844 *	−0.482	0.055	−0.321	0.689	0.683	0.836 *	0.752	0.803	0.697
Circularity	−0.904 *	0.676	0.191	0.500	−0.725	−0.641	−0.712	−0.524	−0.923 **	−0.495

TPN: total pore number, MN: macropore number, CMN: coarse mesoporosity, WC: water content, BD: bulk density, CC: clay content, EC: electrolytic conductivity, OC: organic carbon, TN: total nitrogen, TP: total phosphorus, BN: bacteria number, FN: fungi number, AN: actinomycete number. * Correlation significant at the 0.05 level (2-tailed), ** Correlation significant at the 0.01 level (2-tailed).

## Supplementary Materials

The following are available online at http://www.mdpi.com/1660-4601/15/5/1043/s1, Table S1: Characteristics of sewage effluents and groundwater used for irrigation of agricultural soils, Table S2: Soil physical properties in the S1 irrigated with sewage for 25 years, S2 irrigated with sewage for 52 years, and CK irrigated with groundwater, Table S3: Soil chemical properties in the S1 irrigated with sewage for 25 years, S2 irrigated with sewage for 52 years, and CK irrigated with groundwater, Table S4: Soil microbial properties in the S1 irrigated with sewage for 25 years, S2 irrigated with sewage for 52 years, and CK irrigated with groundwater.
