# Supplementary material for: Effect of Sewage Irrigation on the CT-Measured Soil Pore Characteristics of a Clay Farmland in Northern China
Source: Int J Environ Res Public Health. 2018 May 22;15(5):1043. doi: 10.3390/ijerph15051043 (PMC5982082; doi:10.3390/ijerph15051043)
Supplement: Supplementary file 1 [file ijerph-15-01043-s001.pdf]

International Journal of
Environmental Research and Public Health

MDPI

Supplementary Materials: Effect of Sewage Irrigation on the CT-Measured Soil Pore Characteristics of a Clay Farmland in Northern China

Xiaoming Guo [1], Tongqian Zhao [1,*], Lin Liu [2], Chunyan Xiao [1] and Yuxiao He [1]

Table S1. Characteristics of sewage effluents and groundwater used for irrigation of agricultural soils.

Irrigation water	pH	Electrolytic conductivity (μS cm^{-1})	Total Organic carbon (mg L^{-1})	Na^+ (mg L^{-1})	Ca^{2+} (mg L^{-1})	Mg^{2+} (mg L^{-1})	Sodium adsorption ratio	Cr (μg L^{-1})	Cd (μg L^{-1})	Pb (μg L^{-1})
Sewage effluents	7.7	1599	136.9	162.5	107.7	30.6	3.5	14.9	7.9	25.3
Groundwater	7.2	1347	16.7	46.2	139.8	52.9	0.8	1.1	1.9	2.0

Table S2. Soil physical properties in the S1 irrigated with sewage for 25years, S2 irrigated with sewage for 52 years, and CK irrigated with groundwater.

Sites	Depth (mm)	Water content (%)	Bulk density (g cm^{-3})	Sand content (%)	Silt content (%)	Clay content (%)	USDA textural classification
S1	0-200	21.33±0.10	1.51±0.02	16.45	47.67	35.88	Silty clay loam
	200-400	18.64±0.36	1.68±0.05	17.79	39.00	43.21	Clay
S2	0-200	21.48±0.56	1.61±0.03	26.79	32.67	40.55	Clay
	200-400	18.29±0.31	1.70±0.02	21.45	44.00	34.55	Clay loam
CK	0-200	22.72±0.82	1.57±0.01	23.79	41.33	34.88	Clay loam
	200-400	20.35±0.12	1.61±0.03	22.45	43.33	34.22	Clay loam

USDA: United States; Department of Agriculture, values represent means followed by the standard error.

Table S3. Soil chemical properties in the S1 irrigated with sewage for 25years, S2 irrigated with sewage for 52 years, and CK irrigated with groundwater.

Sites	Depth (mm)	pH	Electrolytic conductivity (μS cm^{-1})	Organic carbon (g kg^{-1})	Total nitrogen (g kg^{-1})	Total phosphorus (g kg^{-1})	Total Chrome (mg kg^{-1})	Total Lead (mg kg^{-1})
S1	0-200	8.58±0.10	268±9.65	24.36±1.09	1.42±0.05	0.71±0.05	16.90±7.84	12.14±4.56
	200-400	8.56±0.06	273±7.68	11.14±0.48	0.71±0.03	0.56±0.01	8.64±5.67	8.96±0.89
S2	0-200	8.37±0.26	286±12.34	24.58±0.75	1.45±0.04	0.82±0.07	32.27±13.99	16.43±6.85

	200-400	0.62±0.40	293±10.02	11.60±0.37	0.58±0.10	0.49±0.03	22.19±2.89	8.39±1.25
CK	0-200	8.64±0.42	173±14.56	19.10±2.20	1.01±0.06	0.68±0.03	6.38±3.92	6.94±0.58
	200-400	0.62±0.06	206±19.06	8.99±0.32	0.61±0.04	0.51±0.06	7.13±1.57	6.77±3.67

Values represent means followed by the standard error.

Table S4. Soil microbial properties in the S1 irrigated with sewage for 25years, S2 irrigated with sewage for 52 years, and CK irrigated with groundwater.

Sites	Depth (mm)	Bacteria number (10^6 cfu g^{-1})	Fungi number (10^4 cfu g^{-1})	Actinomycete number (10^6 cfu g^{-1})
S1	0-200	10.60±5.15	1.08±0.27	9.01±1.74
	200-400	4.09±2.56	0.25±0.00	2.25±0.39
S2	0-200	16.55±1.16	1.06±0.27	13.69±3.50
	200-400	7.33±0.01	0.32±0.19	3.75±0.24
CK	0-200	8.51±1.20	1.17±0.22	5.05±0.71
	200-400	4.25±0.00	0.36±0.27	1.09±0.19

Values represent means followed by the standard error.

© 2017 by the authors. Licensee MDPI, Basel, Switzerland. This article is an open access article distributed under the terms and conditions of the Creative Commons Attribution (CC BY) license (http://creativecommons.org/licenses/by/4.0/).
